# EIF5A regulates proliferation and chemoresistance in pancreatic cancer through the sHH signalling pathway

**DOI:** 10.1111/jcmm.14167

**Published:** 2019-02-14

**Authors:** Zheng Wang, Jie Jiang, Tao Qin, Ying Xiao, Liang Han

**Affiliations:** ^1^ Department of Hepatobiliary Surgery First Affiliated Hospital of Xi’an Jiaotong University Xi’an Shaanxi China; ^2^ Department of Medical Oncology Shaanxi Provincial People’s Hospital Xi’an Shaanxi China

**Keywords:** EIF5A, gemcitabine, pancreatic cancer, proliferation, sHH

## Abstract

**Background:**

Pancreatic cancer (PC) has a very poor prognosis and comparatively short survival. Eukaryotic translation initiation factor 5A (EIF5A) promotes cancer metastasis. Here, we exploited the biological role of EIF5A in PC chemoresistance.

**Methods:**

Expression of EIF5A was analysed in PC cells and tissues by real‐time PCR, Western blotting, immunohistochemistry and immunofluorescent. EIF5A expression was specifically suppressed by transfection, and subsequently the alterations of growth behaviour and resistance to anticancer treatment were tested in an orthotopic tumour model.

**Results:**

The results showed EIF5A was increased in human PC tissues and PC cells. We found EIF5A knockdown reduced the PC proliferation ability in vivo and in vitro. In addition, sonic hedgehog (sHH) signalling pathway may be a downstream of EIF5A in PC cells. Inhibition of EIF5A and sHH signalling pathway could suppress PC cells proliferation and tumour growth. Importantly, EIF5A played an important role in gemcitabine sensitivity for PC.

**Conclusion:**

Taken together, our results revealed that EIF5A regulated the proliferation of PC through the sHH signalling pathway and decreased the Gem sensitivity in PC, which provided a novel therapeutic strategy for PC patients.

## 1 INTRODUCTION

Pancreatic cancer (PC), one of the most lethal and aggressive cancers, has a very poor prognosis and comparatively short survival.^1^, ^2^ Most PC patients are diagnosed at advanced tumour stages partly ascribed to the tumour rapid progression.^3^ Although resection is a unique method for complete cure of PC, it is unusable in most patients due to poor prognosis, late diagnosis and early metastasis.^4^ Therefore, the essential and irreplaceable treatment option available for each PC patient is chemotherapy. Presently, although gemcitabine (Gem) is the most effective chemotherapeutic treatment against PC, unfortunately, Gem was not fully effective on each PC patient because of the resistance to chemotherapy.^5^, ^6^ Therefore, additional treatment options for advanced PC patients are urgently needed.

Eukaryotic translation initiation factor 5A (EIF5A), an 18‐kDa protein, is involved in translation elongation and mRNA transport, and is important for cell proliferation. Vertebrates carry two genes that encode two highly homologous EIF5A isoforms, EIF5A1 and EIF5A2.^7^ To be emphasized, EIF5A2, which implied to be a new oncogene in many types of human cancer as some reports shown,^8^ was focused on in our present study. Previous work showed that EIF5A is up‐regulated in human PC tissues, and inhibition of EIF5A could decrease the PC growth.^9^ In addition, overexpression of EIF5A promotes PC cells metastasis.^10^ The above findings had important implications for the role of EIF5A in PC.

The sonic hedgehog (sHH) signalling pathway plays an important role in pancreas development and differentiation.^11^ sHH, via binding to Patched (PTCH), allows smoothened (SMO) to activate downstream factors such as Gli‐1 and then regulates target gene expression. Presently, the sHH signalling pathway contributes to cancer cell proliferation, differentiation, metastasis and chemotherapy resistance.^12^, ^13^ In the preliminary work, we occasionally found EIF5A could regulate the expressions of sHH and Gli‐1.

Gem, which is a successful compound, frequently is used in PC treatment. Although this drug is effective, its cytotoxic effects and drug resistance limit its application.^14^, ^15^ Such limitation highlights the necessity for exploiting novel treatment strategies which may help overcome drug resistance and enhance tumour cell response to anticancer drugs. In a recent study, the sHH signalling pathway was partly involved in the resistance of PC cells to Gem.^12^ Hence, the objective of this study was to exploit the relationship and molecular mechanisms between EIF5A expression and the activation of sHH signalling pathway in PC. Moreover, we attempted to investigate the biological role of EIF5A in PC chemoresistance in vivo and in vitro.

## 2 MATERIALS AND METHODS

### 2.1 Tissue samples

With the approval and support of the Xi'an Jiaotong University ethics committee, the PC samples from patients (n = 30) with the pathological diagnosis were collected at First Affiliated Hospital of Xi'an Jiaotong University. The normal pancreas tissues were obtained from the donor for liver transplantation. All patients were informed and consent was obtained for the research. The nude mice, provided by animal experiment centre of Xi'an Jiaotong University, were used to construct the animal model. All experimental protocols were approved by the Ethical Committee of the First Affiliated Hospital of Xi'an Jiaotong University, Xi'an, China.

### 2.2 Cell culture and reagents

The human PC cell lines Panc‐1 and BxPc‐3 were obtained from the American Type Culture Collection (ATCC, Manassas, VA, USA) and cultured in DMEM supplemented with 10% foetal bovine serum (FBS) and 1% antibiotic/antimycotic (Life Technologies, Carlsbad, CA, USA). The cells were maintained at 37°C in a humidified 5% CO_2_ atmosphere. Antibodies against sHH (ab53281), SMO (ab5694), PTCH (ab53715), Gli‐1 (ab49314), GAPDH (ab8245) and EIF5A (ab32443) were purchased from Abcam (Cambridge, MA, USA). Recombinant sHH was obtained from R&D Systems (Minneapolis, MN, USA).

### 2.3 Immunohistochemistry

Pancreas samples from patients or nude mice were collected and labelled by antibodies against EIF5A. Each antibody was diluted at a concentration of 1:1000 in 0.1 mol/L phosphate‐buffered saline (PBS) containing 4% normal serum and 0.3% Triton‐X 100 (Sigma). After rinsing with 0.1 mol/L PBS, sections were reacted with biotinylated goat anti‐rabbit IgG (Vector Laboratories, Burlingame, CA, USA) at a dilution of 1:200 in 0.1 mol/L PBS for 2 hours at room temperature. After washing with 0.1 mol/L PBS, they were immersed in a solution of avidin and biotin‐peroxidase complex (Vector Laboratories) at a dilution of 1:100 in 0.1 mol/L PBS for 90 minutes at room temperature. The sections were then immersed in PBS containing 0.1% diaminobenzidine dihydrochloride (Sigma). Antibody‐binding sites were visualized by adding 0.004% hydrogen peroxide. Sections were examined and photographed with a light microscope equipped with digital camera.

### 2.4 Immunofluorescence staining

EIF5A localization in PC cells was examined by immunofluorescence. The prepared cells were washed three times with PBS and then fixed with 100 mL 4% paraformaldehyde in PBS. The cells were permeabilized in blocking buffer (0.1% Triton‐X 100 or 0.1%‐0.5% saponin, 10% NGS, 100 mmol/L PBS, pH 7.4) for 1 hour at room temperature and then incubated with primary antibody to HO‐1 overnight at 4°C. The following day, the cells were washed and incubated with FITC‐conjugated goat antimouse IgG (green fluorescence; 1:500, antimouse, #115165003; Jackson ImmunoResearch, West Grove, PA, USA) for 1 hour at room temperature. The cell nucleus was stained with DAPI (#0100‐20; SouthernBiotech, Birmingham, AL, USA) for 10 minutes. After washing with PBS three times, cells were blocked for 5 minutes. As a negative control, the primary antibody was substituted with antibody diluent.

### 2.5 Transfection

Tumour cells were transfected with EIF5A siRNA. Cells were seeded into small dishes and transfected with 100 nmol/L ShRNA using Lipofectamine 2000 (Invitrogen, Carlsbad, CA, USA) according to the manufacturer's instructions. The cells were used for further experiments 24 hours after transfection. Negative control siRNA (Ambion Inc) was used as a negative control.

### 2.6 Western blotting and real‐time PCR

Western blotting and real‐time PCR were performed as described previously. Briefly, protein was extracted from the cells using lysis buffer [50 mmol/L Tris (pH 7.5), 150 mmol/L NaCl, 1% NP‐40, 0.5% sodium deoxycholate, 1 mmol/L EDTA and 0.1% SDS] containing a protease inhibitor cocktail (Sigma‐Aldrich, St. Louis, MO, USA), and protein concentrations were measured by DC Protein Assay (Bio‐Rad Laboratories Inc, Hercules, CA, USA). Following separation on 7.5% SDS‐polyacrylamide gels, the proteins (20 μL) were transferred onto nitrocellulose membranes (Millipore, Billerica, MA, USA), which were then incubated with the primary antibodies (1:1000) at 4°C overnight. After washing three times with TBST, the membranes were incubated with horseradish peroxidase‐conjugated secondary antibodies. Quantitative analysis was performed with Image‐Pro Plus 6.0 software (Media Cybernetics, Inc, Rockville, MD, USA). The relative protein expression levels were normalized to GAPDH. All experiments were repeated independently three times.

For real‐time PCR, Total RNA was extracted from the cells using TRIzol reagent (Invitrogen), and cDNA was synthesized using a Prime Script RT reagent kit (Takara, Dalian, China). The real‐time PCR experiments were conducted on an iQ5 Multicolor Real‐Time PCR Detection System (Bio‐Rad Laboratories Inc) using SYBRGreen Real‐time PCR Master Mix (Takara). Amplification was carried out as follows: denaturation at 94°C for 3 minutes, 35 cycles of 94°C for 30 seconds, 58°C for 30 seconds and 72°C for 35 seconds. The expression of the target gene was calculated using the 2^−ΔΔCq^ method.

### 2.7 MTT assay

Cell proliferation rate was measured by MTT assays. Briefly, the cells were seeded in 96‐well plates at a density of 1 × 10^4^ cells per well and incubated overnight in medium containing 10% FBS. The DMSO concentration was adjusted to 0.4%. The cells incubated in serum‐free medium were used as the control group. Following incubation for 24, 48 and 72 hours at 37°C, 20 μL of MTT solution (5 mg/mL in PBS) was added to each well, and the cells were incubated for an additional 4 hours at 37°C. Subsequently, 100 μL DMSO was added to each well at 37°C. The optical density (OD) value was determined using a spectrophotometer (Bio‐Rad Laboratories Inc) at 490 nm. The proliferation rate was defined as OD (cell plate)/OD (blank plate).

### 2.8 Orthotopic implantation experiments

Prepared PC cells were injected into the pancreases of nude mice exposed by midline laparotomy (4‐6 sites; 20 μL total volume at a total concentration of 1 × 10^6^/μL). After 4 weeks, the tumour model was highly successful (85%), and drug treatment could be tested. Then, the nude mice were subsequently killed at the indicated time‐points to assess the weight of primary tumours.

### 2.9 Drug treatments

In vitro, recombinant sHH was applied to PC cells at 5 mg/mL. Cyclopamine (Cyc), a sHH pathway inhibitor, was diluted to 18 μg/mL and incubated with cells. The neutralizing antibody of sHH (anti‐sHH, ab171018) was applied to PC cells at 30 μg/mL. Gem was purchased from Sigma‐Aldrich and applied according to the PC cell EC50 (100 μg/mL). All of above were used to cells for 24 hours before being processed. In vivo, recombinant sHH, Cyc and Gem were applied to nude mice at 25, 45 and 125 mg/kg respectively, by intraperitoneal injection.

### 2.10 Statistics

The analyses of the results were carried out using the SPSS statistical software package (version 13.0). The significance of the data was determined using a Student's *t* test. A value of *P* < 0.05 was considered to indicate a statistically significant difference. Data are representative of at least three independent experiments and are reported as means ± SD.

## 3 RESULTS

### 3.1 EIF5A was increased in human PC tissues and PC cells

**Figure 1:**
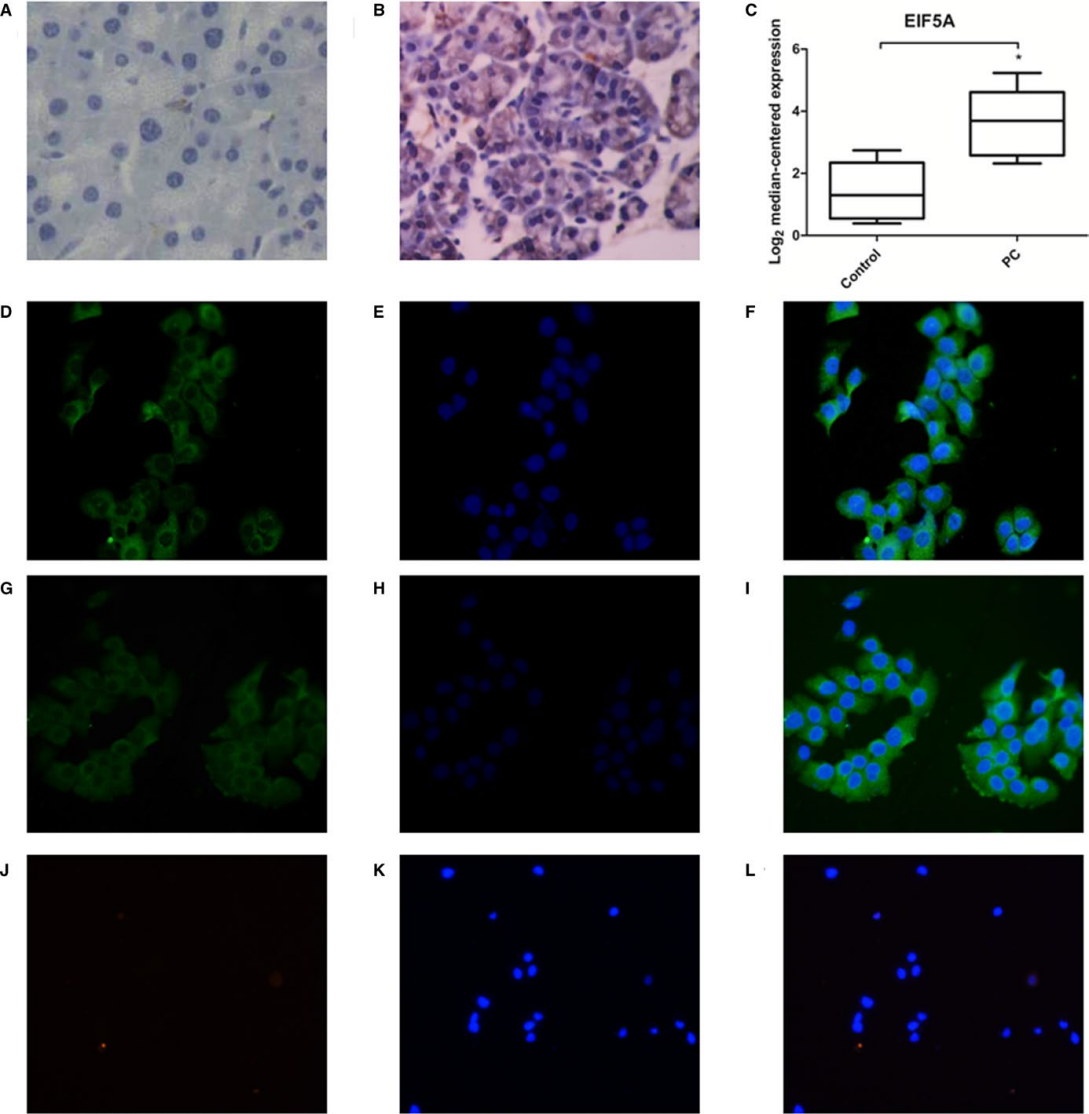
Staining of EIF5A in human PC tissue sections and PC cells. A, Immunohistochemistry staining of EIF5A in human normal pancreatic tissue sections (n = 5). B, Immunohistochemistry staining of EIF5A in human PC tissue sections (n = 30). C, Graphs showing quantitative analyses of EIF5A levels in PC patient samples. D‐I, Immunofluorescence staining of EIF5A in the Panc‐1 and BxPc‐3 cell lines. J‐L, Immunofluorescence staining of EIF5A in pancreatic stellate cells. *P < 0.05, compared with the control (normal pancreatic tissue), as determined by the Student's *t* test

To determine whether EIF5A plays a role in the progression of PC, the expression of EIF5A proteins in normal and PC tissues was immunohistochemically stained with antibody of EIF5A (Figure 1A and B). The results revealed that compared with weak expression of EIF5A in normal pancreatic tissues, PC tissues had overexpression of EIF5A (Figure 1C) (*P* < 0.05). In contrast, we found that the protein expression of EIF5A was identified by the immunofluorescence staining in the Panc‐1 and BxPc‐3 cell lines (Figure 1D‐I). The pancreatic stellate cells were used as a negative control in the research (Figure 1J‐L). These cell experiment results were confirmed in matched pancreatic tissues from PC patients. Thus, the findings suggest that the function of EIF5A may potentially serve to regulate the progression of PC.

### 3.2 Knockdown of EIF5A in PC cells suppressed the PC proliferation ability

**Figure 2:**
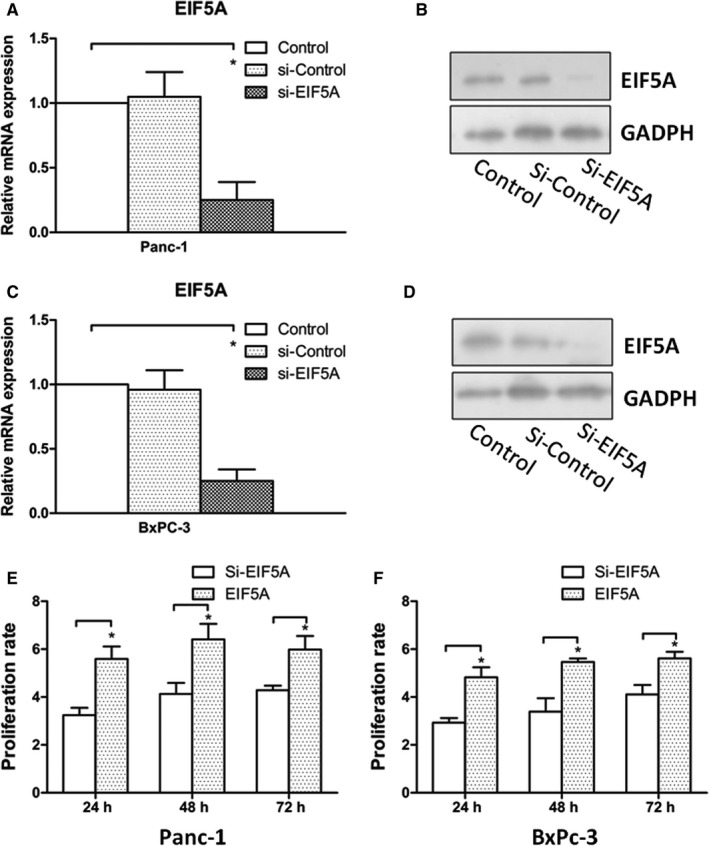
Knockdown of EIF5A suppresses PC cells proliferation in vitro. A, The transfection efficiency of EIF5A knockdown in Panc‐1 cells was verified by real‐time PCR. B, The transfection efficiency of EIF5A knockdown Panc‐1 cells was examined by Western blot analysis, which revealed similar results with real‐time PCR. C and D, The transfection efficiency of EIF5A knockdown in BxPc‐3 cells was verified by real‐time PCR and Western blot analysis. E, The effects of EIF5A on Panc‐1 cells and proliferation were determined by MTT assay. (Mean±SD 3.24 ± 0.3130, 4.13 ± 0.4630, 4.28 ± 0.1939 at 24, 48 and 72 h for Si‐EIF5A groups. Mean±SD 5.59 ± 0.5200, 6.41 ± 0.6500, 5.98 ± 0.5700 at 24, 48 and 72 h for EIF5A groups; n = 6.) F, The effects of EIF5A on BxPc‐3 cells proliferation were determined by MTT assay. (Mean±SD 2.93 ± 0.1930, 3.39 ± 0.5630, 4.11 ± 0.3939 at 24, 48 and 72 h for Si‐EIF5A groups. Mean±SD 4.82 ± 0.4200, 5.46 ± 0.1500, 5.62 ± 0.2700 at 24, 48 and 72 h for EIF5A groups; n = 6.) The data showed knockdown of EIF5A suppresses PC cells proliferation. *P < 0.05 as determined by the Student's *t* test

To determine whether EIF5A plays an important role in the PC cells proliferation ability, the Panc‐1and BxPc‐3 cells were prepared for transfection with or without stable EIF5A knockdown using ShRNA. The transfection efficiencies were proved by real‐time PCR (Figure 2A and C) and Western blotting analysis (Figure 2B and D). Thus, the new transfected PC cells, with approximately 90% decrease in EIF5A protein levels, were marked as Si‐EIF5A, in order to carry out the subsequent research.

The cell proliferation was measured by MTT assays at 24, 48 and 72 hours following with or without transfection. We found that the proliferation ability was significantly reduced upon EIF5A knockdown compared to control group (Figure 2E and F) (*P* < 0.05). In addition, the reduced proliferation ability kept the consistent results at 24, 48 and 72 hours. Partially, these findings suggested the importance of EIF5A and it played a role in PC cells proliferation ability.

### 3.3 PC cells with Si‐EIF5A expression showed decreasing tumour growth in in situ tumour model

**Figure 3:**
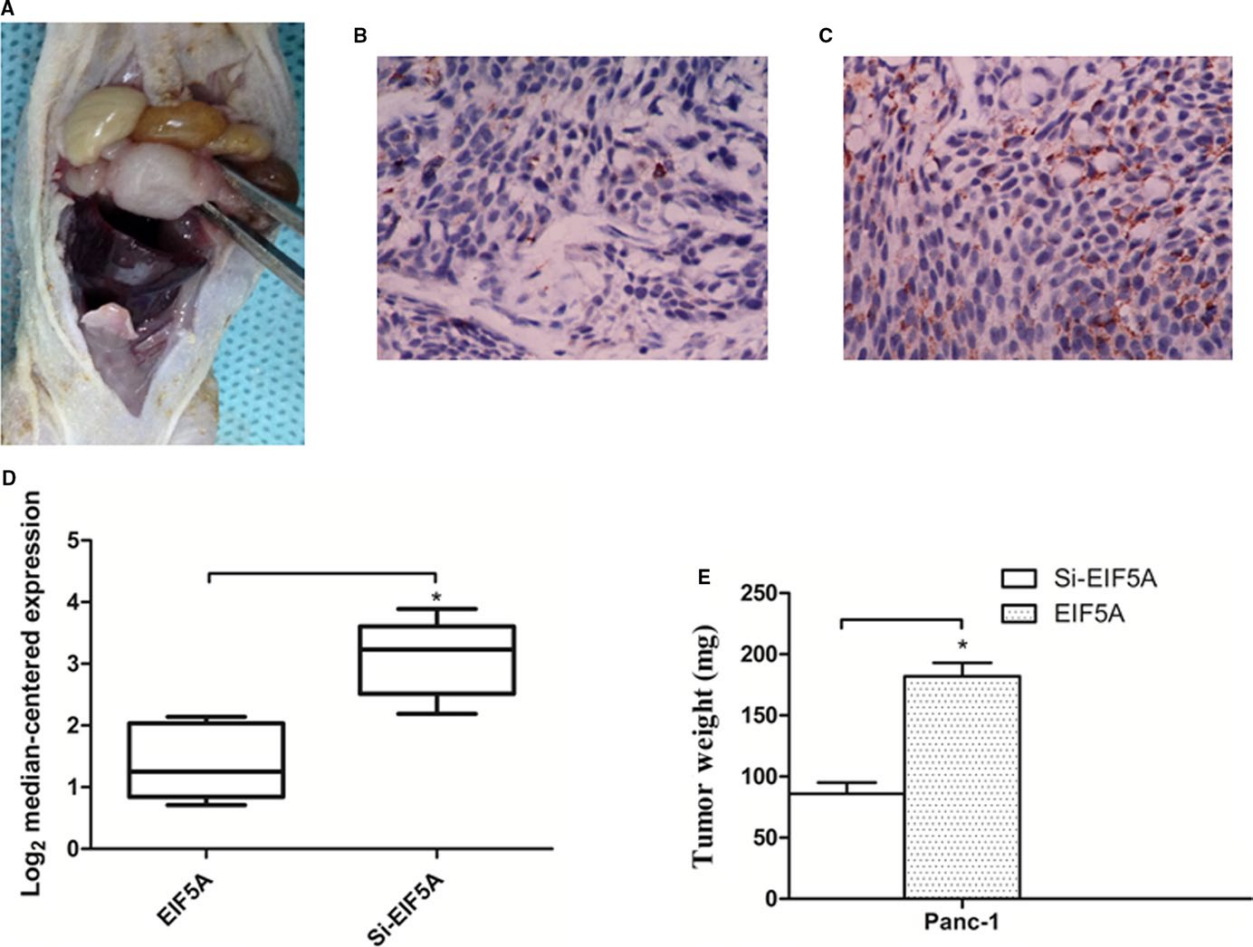
Knockdown of EIF5A shows increasing tumour growth in situ tumour model in vivo. A, Prepared Panc‐1 cells were injected into the pancreases of nude mice exposed by midline laparotomy. After 4 wk, the tumour model was successful. Immunohistochemical staining showed the expression of EIF5A in tumour model with normal Panc‐1 cells (B), or Panc‐1 cells with Si‐EIF5A (C). D, Graphs showed quantitative analyses of EIF5A levels in tumour model samples. E, Tumour size in mice was determined by tumour weight. *P < 0.05 as determined by the Student's *t* test. (n = 8 for each group.)

To confirm the cell proliferative potential caused by EIF5A in vitro experiment, we designed an in situ tumour model in nude mice (Figure 3A). Panc‐1 cells, with or without transfection, were injected into the pancreases of nude mice. After 4 weeks, we measured the tumour size by assessing the weight of primary tumours in mice.

We sought to verify the expression of EIF5A in tumours through immunohistochemically stained using EIF5A antibody. The results showed weak expression of EIF5A in the group of Panc‐1 cells with Si‐EIF5A in tumour model (Figure 3B). In contrast, the normal Panc‐1 cells had overexpression of EIF5A protein (Figure 3C). Obviously, there was significant difference in EIF5A levels between the two groups (Figure 3D) (*P* < 0.05). The Panc‐1 cell group formed significantly larger tumour size in vivo compared with Panc‐1 cells with Si‐EIF5A (Figure 3E) (*P* < 0.05). Taken together, these findings demonstrated that down‐regulation of EIF5A prevented proliferation ability in PC progress.

### 3.4 EIF5A regulated sHH signalling pathway in PC cells

**Figure 4:**
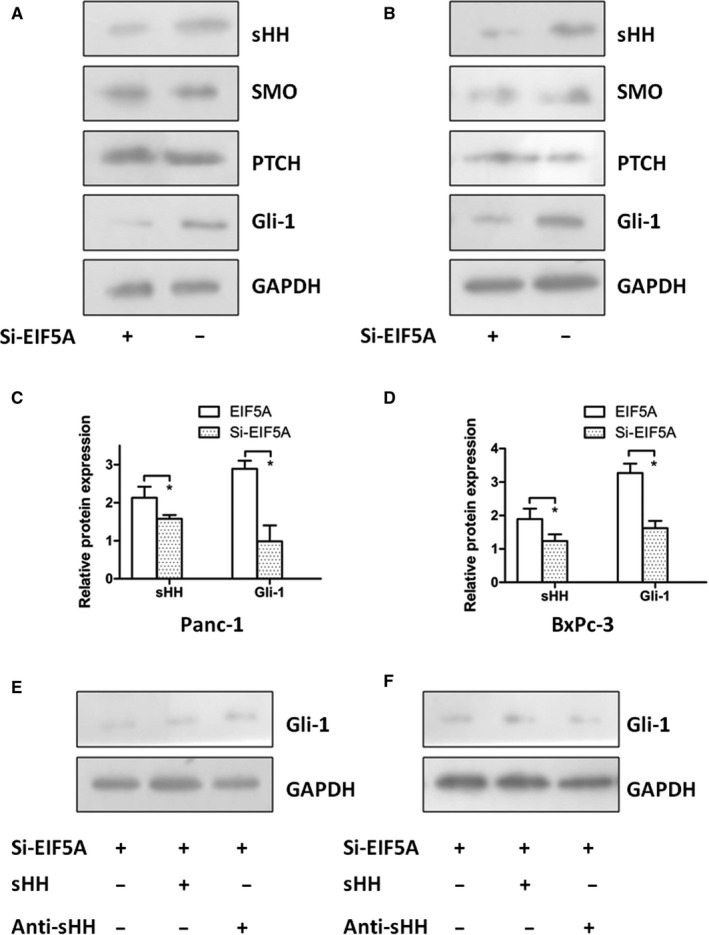
The expression of EIF5A controls the sHH signalling pathway in PC cells. The Western blotting showed that EIF5A knockdown can decrease the protein expression of sHH signalling factors in Panc‐1 cells (A) and BxPc‐3 cells (B). Graphs showed the EIF5A knockdown obviously reduced the expressions of sHH and Gli‐1 in Panc‐1 cells (C) and BxPc‐3 cells (D). To decide whether activation of sHH signalling pathway depends on sHH factor, we used recombinant sHH or neutralizing antibody to treat the PC cells with Si‐EIF5A. The Western blotting results showed the low expression level of Gli‐1 for Panc‐1 cells (E) and for BxPc‐3 cells (F). *P < 0.05 as determined by the Student's *t* test

To determine whether sHH signalling pathway is the downstream effector of EIF5A in PC cell proliferation, firstly, we investigated whether the EIF5A knockdown can decrease the protein expression of sHH signalling factors in different PC cell lines (Figure 4A and B). As shown in Figure 4, Western blotting was used to quantify protein levels, and the EIF5A knockdown obviously reduced the expressions of sHH and Gli‐1 in Panc‐1 and BxPc‐3 cells (Figure 4C and D) (*P* < 0.05), but we did not observe differential expression of SMO and PTCH in any group (data not shown) (*P* > 0.05). Obviously, these results showed that EIF5A activated the sHH signalling pathway in PC cells. But whether the activation of sHH signalling pathway depends on sHH factor, another experiment was designed, which used recombinant sHH or neutralizing antibody to treat the PC cells with Si‐EIF5A. Then, the protein expression of Gli‐1 was measured by Western blotting. The results showed that Gli‐1 remained low expression in PC cells containing Si‐EIF5A (Figure 4E and F) (*P* < 0.05). Altogether, these data indicated that sHH signalling pathway may be a downstream of EIF5A but the sHH factor, independent of sHH canonical stimulation.

### 3.5 Inhibition of EIF5A expression and sHH signalling pathway suppressed PC cells proliferation and tumour growth

**Figure 5:**
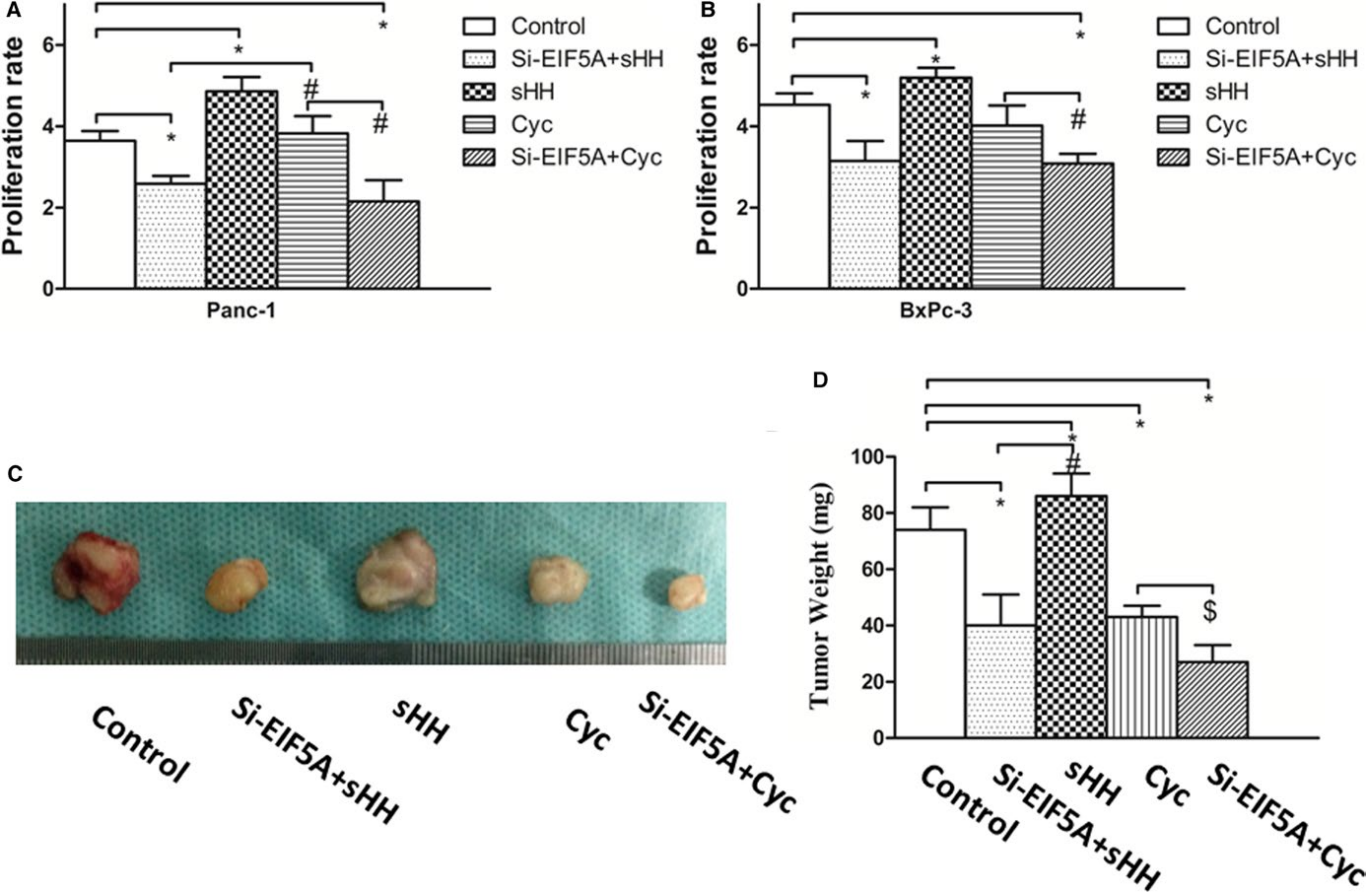
Inhibition of EIF5A and sHH signalling pathway decreases PC cells proliferation and tumour growth. A, The MTT results showed that the Panc‐1 cells treatment with Si‐EIF5A combined using Cyc obviously decreased the proliferative ability. (Mean±SD 3.640 ± 0.240 for control, 2.590 ± 0.19 for Si‐EIF5A+sHH group, 4.860 ± 0.350 for sHH group, 3.825 ± 0.425 for Cyc group.) B, The MTT results showed that the BxPc‐3 cells treatment with Si‐EIF5A combined using Cyc obviously decreased the proliferative ability compared with other groups (Mean±SD 4.532 ± 0.280 for control, 3.147 ± 0.49 for Si‐EIF5A+sHH group, 5.192 ± 0.250 for sHH group, 4.019 ± 0.492 for Cyc group.) sHH could significantly increase cells proliferation. *P < 0.05, compared with control, ^#^ *P* < 0.05, compared with the Cyc group. C, Tumour size in mice was measured by the tumour weight, and the sHH group showed a strongly increasing tumour growth, but the group of Si‐EIF5A and Cyc obviously decreases the tumour growth. D, Graphs showed that Si‐EIF5A and sHH signalling could affect the tumour weight. *P < 0.05, compared with the control, ^#^ *P* < 0.05, compared with the sHH group, ^$^ *P* < 0.05, compared with the group of Si‐EIF5A and Cyc

Our above work showed that EIF5A regulated Gli‐1 protein expression in PC cells. To determine the effect of EIF5A and sHH signalling pathway for PC cells proliferation, the Panc‐1 and BxPc‐3 cells with Si‐EIF5A were treated with recombinant sHH, or Cyc which is a sHH signalling pathway inhibitor. As shown in Figure 5A and B, the results revealed that treatment with sHH significantly increased cells proliferation, but the Si‐EIF5A combined using Cyc could most obviously decrease the proliferative ability in comparison with control or the other intervention groups (*P* < 0.05). Additionally, the cancer cells with Si‐EIF5A combined using recombinant sHH could inhibit the proliferation significantly (*P* < 0.05). Hence, these results demonstrated that EIF5A and sHH signalling pathway may be co‐involved in PC cells proliferation.

To determine the effect of EIF5A and sHH signalling on PC tumour growth in vivo, we assessed orthotopic tumour formation of Panc‐1 cells with Si‐EIF5A. After 4 weeks, the cells were treated with recombinant sHH, or Cyc, then, the average tumour mass of different cell groups was measured (Figure 5C). A similar result was also observed in tumour size, compared with PC cells proliferation. The sHH group showed a strongly increasing tumour growth, but the group of Si‐EIF5A and Cyc displayed the most obviously adverse effect on tumour size (Figure 5D) (*P* < 0.05). Collectively, these results demonstrated that EIF5A and sHH signalling pathway was sufficient and necessary for tumour growth in PC.

### 3.6 The expression of EIF5A and activation of sHH signalling pathway were regulated by Gem in PC cells in vitro

**Figure 6:**
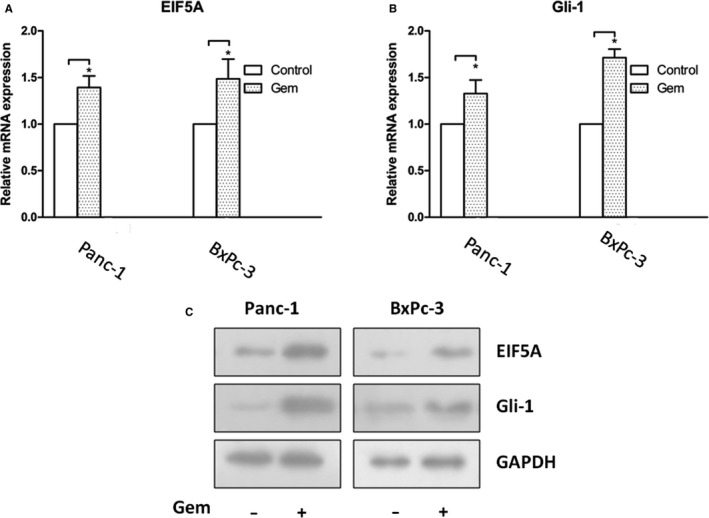
EIF5A protein expression and sHH signalling are changed by Gem in PC cells. The quantification data from real‐time PCR revealed that Gem significantly increased the mRNA expression of EIF5A (A) and Gli‐1 (B) compared with control group in Panc‐1 and BxPc‐3 cells. C, The Western blotting showed that Gem significantly enhanced EIF5A and Gli‐1 protein expression in both cells. *P < 0.05, compared with control (without Gem group)

To validate whether the EIF5A protein expression and sHH signalling changes is detected by Gem, we treated the PC cells with an EC50 dose of Gem for 24 hours. The quantification data from real‐time PCR revealed that Gem significantly increased the mRNA expression of EIF5A compared with control group in Panc‐1 and BxPc‐3 cells (Figure 6A) (*P* < 0.05). Additionally, we examined the expression of sHH signalling factors in the PC cells treated with Gem. Similarly, Gem increased the expression of Gli‐1 compared with control (Figure 6B) (*P* < 0.05). However, Gem up‐regulated sHH expression, but did not reach statistical significance (data not shown) (*P* > 0.05). Then, we investigated whether Gem regulate the EIF5A and sHH signalling expression in protein level. Western blotting was used to quantify protein levels, and the results showed that Gem significantly enhanced EIF5A and Gli‐1 protein expression in Panc‐1 and BxPc‐3 cells (Figure 6C) (*P* < 0.05). Therefore, these data from mRNA and protein analyses indicate that EIF5A may be involved in the sensitivity of Gem in PC cells.

### 3.7 Targeting EIF5A increased Gem sensitivity in PC in vitro and in vivo

**Figure 7:**
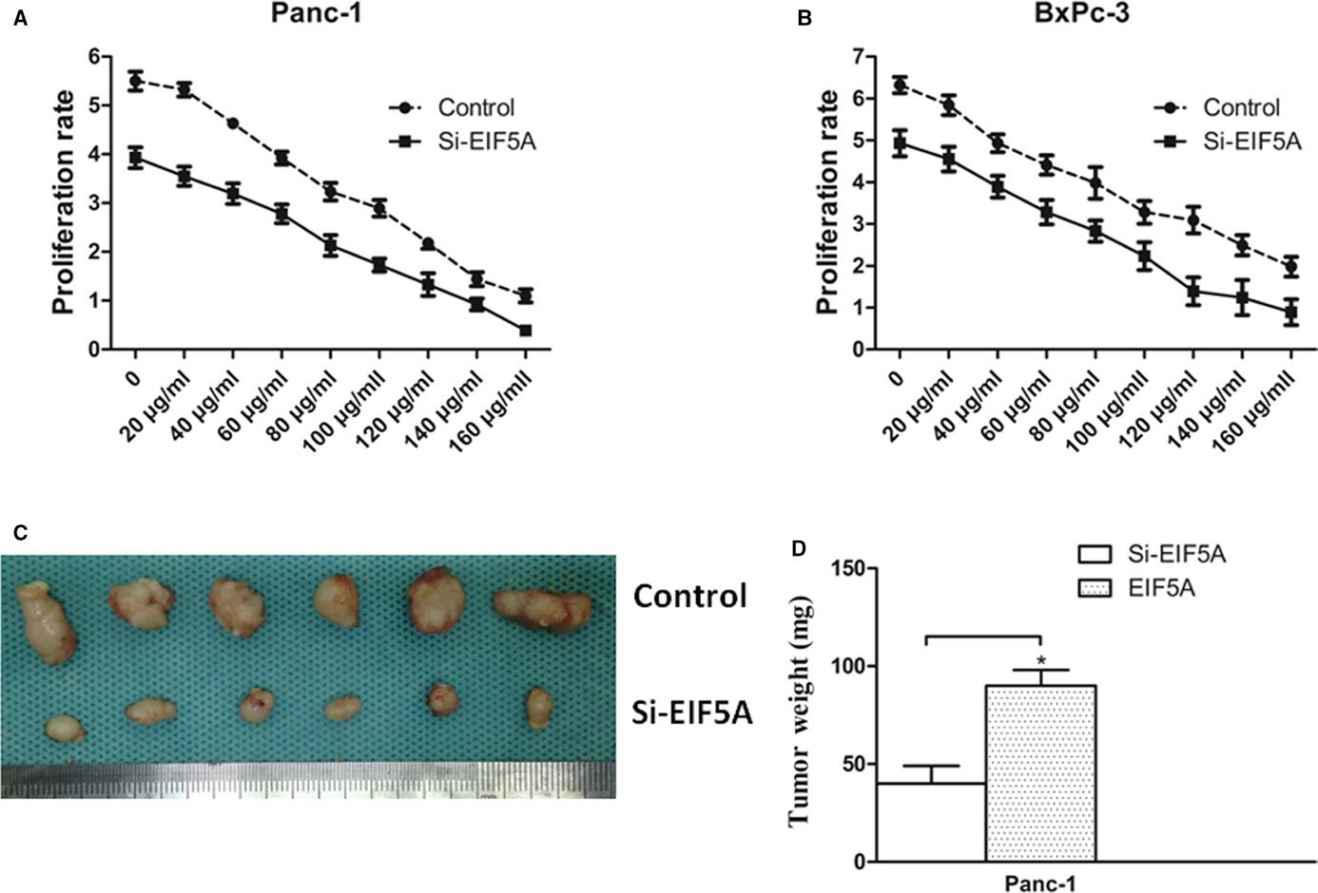
Inhibiting EIF5A increases Gem sensitivity. Proliferation ability of Panc‐1 (A) and BxPc‐3 cells (B) showed that the cell proliferation was dose‐dependent on the concentration of Gem, and the PC cells with Si‐EIF5A exhibited an obviously decreased proliferative ability compared with control group. C, Panc‐1 cells treated only with Gem showed a larger tumour size compared with Si‐EIF5A group. D, Graphs showed knockdown of EIF5A with Si‐EIF5A obviously decreased the tumour growth. *P < 0.05 as determined by the Student's *t* test (n = 8 for each group.)

In our previous work, we found that Gem leaded the up‐regulation of EIF5A expression in PC cells. So, we hypothesized that EIF5A could affect the sensitivity of Gem. Then, we examined PC cell proliferation ability when Panc‐1 and BxPc‐3 cells, with or without the Si‐EIF5A, were treated with different concentrations of Gem for 24 hours. As shown in Figure 7A and B, the results of the dose‐dependent experiments of Gem treatment were shown. We found that the PC cells transfected with the Si‐EIF5A exhibited an obviously decreased proliferative ability compared with control group (*P* < 0.05). This phenomenon supported that EIF5A contributed to Gem sensitivity in PC cells. Furthermore, we confirmed the correlation between EIF5A and Gem sensitivity through orthotopic tumour formation of Panc‐1 cells in vivo (Figure 7C). Indeed, the Panc‐1 cells treated only with Gem showed a larger tumour size compared with Si‐EIF5A group (Figure 7D) (*P* < 0.05). These findings indicated that EIF5A played an important role in Gem sensitivity for PC and suggested that combination therapies involving Gem and EIF5A might benefit PC patients.

## 4 DISCUSSION

Pancreatic cancer remains one of the most aggressive malignancies, because of its poor prognosis, late diagnosis and rapid dissemination, with less than 7% survival at 5 years.^1^ Most PC patients are detected at an advanced stage due to the difficulty of early diagnosis. A number of proliferative promoters induce PC rapid progression.^16^ Because tumour growth is based on augmented cell growth and prolonged cell survival, the treatment options for growth inhibitory adjuvant to traditional therapy, such as surgical resection, radiotherapy and chemotherapy, are urgently requisite. Currently, chemotherapy is not effective for every PC patient at all stage of treatment.^17^, ^18^ Although Gem is the most effective chemotherapeutic treatment against PC, its efficiency keeps in a lower rate.^19^, ^20^

It was known that EIF5A was involved in transcription, mRNA turnover and nucleocytoplasmic transport in cells. Usually, it has two EIF5A isoforms, EIF5A1 and EIF5A2. EIF5A1 is the major isoform which is abundantly expressed in most cells.^8^ EIF5A2 is expressed in few normal tissues but is overexpressed in tumour cells and even considered a candidate oncogene.^21^ Based on above, EIF5A2 is chosen as our research focus in the study instead of EIF5A1. Important recent work has shown that EIF5A2 was high expression in PC and regulated the mechanisms of pathogenesis and metastasis.^16^, ^22^ Therefore, in this work, the relationship and mechanism of EIF5A2 impacting with PC proliferation were discussed. Here, the results provided evidence that EIF5A was a major regulator controlling proliferation and chemosensitivity.^23^

Sonic hedgehog is abnormally expressed in PC tissue and cells and associated with pathogenesis and progression. sHH is one of the three members family of hedgehog proteins, which includes other two proteins named as Indian Hedgehog and Desert Hedgehog. Binding of hedgehog proteins to the transmembrane receptor Patched activates SMO, leading to nuclear translocation of Gli transcription factors and expression of downstream target genes.^24^, ^25^ A report showed that sHH signalling pathway could promote the pancreatic desmoplasia in PC cells.^26^ As reported previously, sHH regulated pancreatic fibrosis and cancer cell proliferation and differentiation.^27^, ^28^ We recently identified that sHH signalling pathway played a role in PC progress as a regulator to chemosensitivity.^12^ Hence, inhibition of sHH signalling may be an attractive clinical target for therapeutic intervention.

The data presented here demonstrated an important role for EIF5A in regulating PC proliferation^8^, ^17^ in vitro and in vivo, and the inhibition of EIF5A enhanced the responsiveness of colorectal cancer to Gem.^18^ Also, sHH signalling pathway was involved in the regulating process.

We reported that high expression of EIF5A protein was observed in PC. The immunohistochemical analyses demonstrated the up‐regulation of EIF5A in PC tissues compared with normal pancreatic tissues. The immunofluorescence staining showed that EIF5A was identified in PC cells. Our found had the consistent aspect with that EIF5A protein was amplified in many neoplastic patient tissues.^16^, ^29^, ^30^ The results suggested that EIF5A was involved in PC and may be a critical contributor to PC progression. Understanding the expression of EIF5A in PC is the study foundation for next research.

Then, the Panc‐1 and BxPc‐3 cells were transfected for stable knockdown EIF5A using shRNA. MTT results showed that the proliferation ability was significantly reduced upon EIF5A knockdown compared to control group. In fact, the reduced proliferation ability kept similar result at every time‐point. Thus, these findings suggested that EIF5A played a role in PC cells proliferation ability, which was consistent with the idea that EIF5A contributes to tumour growth in other cancers.^23^ Of cause, the MTT assays are not sufficiently convincing for promoting cell proliferation caused by EIF5A. Thus, we made further experiment in vivo to check the above results.

In this work, in order to investigate the tumour growth caused by EIF5A, we built the orthotopic transplantation tumour model in nude mice with PC cells, instead of the subcutaneously implanted tumour model. Mainly because the PC cells were injected into the pancreas of nude mice being more like the tumour microenvironment.^31^ The immunohistochemical analyses showed only weak expression of EIF5A in the group with Si‐EIF5A in tumour models compared with Panc‐1 cells group. So, the necessary step proved the success of the orthotopic model with different expression of EIF5A in tissue level. These findings suggested that the Panc‐1 cell group formed significantly larger tumour size compared with Panc‐1 cells with Si‐EIF5A, which had the similar results with one in vitro.

Our previous work showed sHH signalling pathway was involved in the PC growth. Here we demonstrated that EIF5A induced the activation of the sHH signalling pathway. The EIF5A knockdown obviously reduced the expressions of sHH and Gli‐1 in PC cells. To decide whether the activation of sHH signalling pathway depends on sHH factor, we used recombinant sHH or neutralizing antibody to treat PC cells with Si‐EIF5A. The results showed low expression of Gli‐1 in Si‐EIF5A group. Altogether, these data indicated sHH factor was unnecessary in activation caused by EIF5A. Possibly the bypass activation or other transcriptional activation ways could be involved. The internal mechanism will be solved in further study.

In following experiments, the growth results showed that sHH significantly increased cells proliferation, and the Si‐EIF5A obviously decreased the proliferative ability. Together, these results demonstrated that EIF5A and sHH signalling pathway may be involved in and were necessary for tumour growth in PC, which is consistent with our pre‐primary work.

Gem is frequently used in PC treatment. However, it is only marginally effective because of the less sensibility.^32^ In this study, Gem significantly increased the expression of EIF5A and Gli‐1 in PC cells. The Si‐EIF5A exhibited an obviously decreased proliferative ability when PC cells were treated with Gem. In vivo, the Panc‐1 cells treated only with Gem showed a larger tumour size compared with Si‐EIF5A group. These findings indicated that EIF5A played an important role in Gem sensitivity for PC. Therefore, these data imply that combination therapies involving Gem and EIF5A might be a promising strategy for PC.

In summary, our results revealed that EIF5A regulates the proliferation in PC through the SHH signalling pathway. Modulating the expression level of EIF5A could enhance the Gem sensitivity in PC. Importantly, the target spot to EIF5A and SHH signalling pathway could benefit PC patients in future.

## CONFLICTS OF INTEREST

The authors declare that there are no conflicts of interest related to this work.
